# Mental Health of Residents of Ukraine Exposed to the Russia-Ukraine Conflict

**DOI:** 10.1001/jamanetworkopen.2024.59318

**Published:** 2025-02-13

**Authors:** Jiafu An, Tenghui Wang, Bin Chen, Anatoly Oleksiyenko, Chen Lin

**Affiliations:** 1 Faculty of Architecture, The University of Hong Kong, Hong Kong SAR; 2 Faculty of Business, Lingnan University, Hong Kong SAR; 3 Department of International Education, Education University of Hong Kong, Hong Kong SAR; 4 Faculty of Business and Economics, The University of Hong Kong, Hong Kong SAR

## Abstract

**Importance:**

Quantifying the association between the ongoing Russia-Ukraine war and Ukrainians’ mental health is crucial for guiding reconstruction and rebuilding efforts in the postwar period.

**Objective:**

To examine the association between war exposure and civilians’ mental health in both the short and medium term and the potential channels through which these associations occur.

**Design, Setting, and Participants:**

This cohort study used data from 7 nationally representative surveys conducted by the Kyiv International Institute of Sociology between December 4, 2015, and July 18, 2022. Six surveys were conducted before the war and one during the ongoing conflict in July 2022. Each survey included approximately 2000 respondents aged 18 to 95 years in 110 localities.

**Exposure:**

The magnitude of war damage exposure was represented by an indicator variable set to 1 if an oblast (province) experienced severe war damage and 0 if the damage was moderate at the time of the survey. The classification of severe or moderate damage was based on the economic losses incurred.

**Main Outcomes and Measures:**

The main outcomes were respondents’ self-reported thoughts of suicide and feelings of helplessness, both represented as dummy variables.

**Results:**

The sample included 14 140 respondents, with 3933 (27.8%) exposed to severe war damage and 10 207 (72.2%) to moderate war damage. These included 8174 female respondents (57.8%), with an overall mean (SD) age of 48.9 (16.8) years. The differences in the likelihood of reporting suicidal thoughts and feelings of helplessness between areas with severe vs moderate war damage increased by 2.2 percentage points (β estimate, 0.022 [SE, 0.006]; 95% CI, 0.009-0.036) and 4.9 percentage points (β estimate, 0.049 [SE, 0.019]; 95% CI, 0.009-0.088), respectively. Subsample analyses revealed that male respondents were more vulnerable to suicidal thoughts (β estimate, 0.030 [SE, 0.013]; 95% CI, 0.004-0.056), while female respondents were more at risk of feelings of helplessness (β estimate, 0.063 [SE, 0.024]; 95% CI, 0.013-0.113).

**Conclusions and Relevance:**

In this survey study of war exposure, bombing was associated with increased mental health concerns among civilians, including a higher likelihood of suicidal thoughts and feelings of helplessness, which may hinder postwar recovery. These findings suggest that policymakers should prioritize creating job opportunities and ensuring a safe living environment to support citizens’ recovery in the aftermath of conflict.

## Key Points

**Question:**

What is the association between war exposure and civilians’ mental health during the ongoing Russia-Ukraine conflict?

**Findings:**

This survey study, using data from 7 nationally representative surveys (2015-2022) with 14 140 respondents, found that severe war damage was associated with significantly increased likelihood of suicidal thoughts by 2.2 percentage points and feelings of helplessness by 4.9 percentage points compared with moderate damage. Individuals with higher levels of wealth, male respondents (for suicidal thoughts), female respondents (for helplessness), respondents with higher levels of education, and younger individuals were particularly vulnerable.

**Meaning:**

These findings suggest that war exposure was associated with significant mental health challenges.

## Teaser

This cohort study uses rates of suicidal thoughts and feelings of helplessness to assess the association between population mental health and the ongoing conflict between Russia and Ukraine.

## Introduction

War leaves profound and enduring adverse effects on economic development.^1,2^ While many estimates focus on the physical impacts of war,^1,3^ they reveal a puzzling pattern: although recovery from the direct physical damages of wars can be relatively quick,^4,5^ the economic costs of war can reach up to 18% of the gross domestic product per capita^3^ and last as long as 20 years.^6,7^ A critical yet often overlooked aspect in these evaluations is the impact of mental health disorders, which not only contribute to the global burden of disease but also hinder postwar economic and societal recovery.^8,9^ These disorders are leading contributors to preventable mortality and long-term disability, deserving advanced health care planning and resource allocation. This underscores the urgent need to address mental health as a central component of postconflict recovery efforts.

Although existing research, largely based on postconflict surveys, provides valuable insights, the results are often confounded by time-invariant locational factors that are difficult to control for.^10,11,12,13,14^ Notable exceptions^15,16,17^ use novel identification strategies, such as the quasi-random assignment of military personnel to combat vs noncombat zones, to address omitted variable bias. However, their attention is often constrained by the particular settings and thus limited to service members, immigrants,^18^ and/or veterans. While these studies provide insight, they do not study the psychological impact of war on the broader civilian population affected by the conflict. Filling this research gap is vital, as the mental health of the general public is central to the reconstruction and rebuilding efforts in the postwar period.^19,20,21,22^ In this regard, recent contributions in the medical literature emphasize the substantial burden of mental disorders globally, with studies demonstrating their important role in mortality, disability, and long-term health outcomes.^23,24^ By integrating these perspectives into the study of the mental health impact of war, we aim to provide a more comprehensive understanding of the issue and complement this line of research.

In this cohort study, we examined the associations between population mental health and the ongoing war between Russia and Ukraine, which began with the Russian invasion in 2022. The background of the conflict is shown in eAppendix 1 in Supplement 1. The war has caused substantial damage to Ukraine.^25,26,27^ Despite these extensive losses, there is limited understanding of how the conflict is associated with the mental health of the Ukrainian population.^28^

We focused on 2 key issues: assessing the association between mental health and the war using difference-in-differences (DID) methodology and identifying subsamples that are particularly vulnerable to the war’s effects, which can inform humanitarian intervention efforts. In eAppendix 1 in Supplement 1, we provide a background of the recent Russian invasion of Ukraine.

## Methods

This cohort study used existing, deidentified archival data and presented low risk. Informed consent was obtained from all participants when the Kyiv International Institute of Sociology began conducting the interviews. We reported the information to the Sub-Committee on Research Ethics and Safety of the Research Committee of Lingnan University. After its confirmation, the board deemed this study exempt from ethics review and waived the informed consent requirement because the study used existing, deidentified archival data and presented low risk. We followed the Strengthening the Reporting of Observational Studies in Epidemiology (STROBE) reporting guideline.

### Sample

We obtained 7 nationally representative surveys conducted by the Kyiv International Institute of Sociology from December 4, 2015, to July 18, 2022. This dataset includes 6 surveys administered prior to the outbreak of the war and one conducted during the ongoing conflict (in July 2022). Each survey involved approximately 2000 respondents aged 18 to 95 years in 110 localities across all 25 Ukrainian oblasts (provinces), covering demographic characteristics and mental health conditions.

### Measures

#### Mental Health

Suicidal ideation is a widely used measure of mental health disorders and is incorporated into various mental illness screening tools, such as the clinically validated Patient Health Questionnaire–9^29,30^ and the Suicide Behaviors Questionnaire–Revised.^31^ Research indicates that thoughts of suicide are associated with actual suicidal behavior and completed suicide.^32,33^ Learned helplessness, a state in which people believe that they are unable to control or change a situation even when they have the ability to do so, is often seen in cases of depression^34^ and is a common precursor to severe mental disorders.^35^ Both measures have been frequently used to examine mental health conditions following violent events.^13,36,37^

Based on the information provided in the survey, we constructed 2 key measures of mental health. Suicidal thoughts or learned helplessness were scored as 1 if a respondent answered “yes” to the question “Have you lived in a state close to suicide/felt helpless during the year” and 0 otherwise.

#### War Damages Exposure

The war damages exposure is an indicator that equals 1 if the oblast experienced severe war damages and 0 if the damage was moderate. We defined exposure in 2 ways. First, we considered oblasts to have severe war damage if the value of the losses exceeded $5 billion (ie, the median value of damages of the affected oblasts) by June 1, 2022, as estimated by official institutions,^25^ and others to have moderate damages.

We also changed the threshold from $5 billion to $7.5 billion, with a $1 billion loss, the mean or median level of attacks experienced since the war broke out, to verify the robustness of the results. Moreover, to make a sharper comparison and hence more clearly estimate the impact of the war, we changed the threshold from $5 billion to $10 billion, and to ensure that these places were similar, we further restricted areas classified as having moderate war damage to those adjacent to areas with severe damage.

#### Other Variables

We also constructed several variables based on the individual characteristics such as sex, age, educational level, nationality, occupation, and wealth (ie, self-reported low, middle, and high income), as well their experiences of losing jobs, surviving bombing, being without a livelihood, and surviving the death of loved ones. We defined these variables in detail in eTable 1 in Supplement 1 and presented summary statistics in eTable 2 in Supplement 1 for the full sample and eTable 3 in Supplement 1 for the July 2022 wave survey.

### Statistical Analysis

We first assessed whether the outbreak of the Russia-Ukraine war was associated with increased mental health distress among Ukrainian residents. There is a possibility that a common, third factor contributed to both the deterioration of mental health and the outbreak of the war, which would preclude us from drawing definitive inferences from the time series patterns alone. Therefore, we applied a DID approach that allows us to control for time-invariant factors of the attacked places. In particular, we compared the changes of Ukrainians’ mental health conditions before and after the outbreak of the war between places that experienced severe vs moderate war damages. We used the following ordinary least squares model in Stata, version 17 (StataCorp LLC), to estimate the association between exposure to war and mental health:Mh_ijt_ = βDamage_ij_ × Post_it_ + α_1_Damage_ij_ + α_2_Post_it_ + α_3_Controls_ijt_ + FEs + ε_ijt_, where MH_ijt_ is either suicidal thoughts or helplessness for respondent i in oblast j from survey wave t. Damage_ij_ is an indicator representing the magnitudes of war damages. Post_it_ equals to 1 if respondents were surveyed after the outbreak of the war on February 24, 2022, and 0 otherwise. Controls_ijt_ include respondents’ sex, age, age-squared, educational level, nationality, and occupation. We included oblast and survey wave as fixed effects to account for oblasts’ unobserved time-invariant factors and common time-related factors. ε_ijt_ is the residual term. We clustered the SE at the oblast level.

The core identifying assumption of the DID approach is that locations with varying levels of war exposure were following parallel trends in mental health conditions prior to the war’s outbreak. Theoretically, we argue that this assumption is plausible since Russia’s initial military strategy was to seize Kyiv quickly, rather than targeting mentally healthy populations. Empirically, we conducted a dynamic DID analysis to examine whether the parallel trend assumption holds in our case. Specifically, we decomposed the prewar periods and interacted the dummies variables with an indicator of the extent of war damages, while controlling for oblast and survey fixed effects, and clustering SEs at the oblast level. Two-sided *P* < .10 (10% level) indicated statistical significance.

We also investigated the association between Russia’s bombing and people’s mental health in the very short term using an alternative strategy. During the period from July 6 to July 18, 2022, several survey locations were bombed. This created random exposure to the bombing for people interviewed before and after the event, allowing us to study the short-term association between the war and mental health. We introduce this strategy in detail in eAppendix 2 in Supplement 1.

## Results

### War, Suicidal Ideation, and Learned Helplessness

The sample included 14 140 respondents, of whom 3933 (27.8%) experienced severe war damage and 10 207 (72.2%) were exposed to moderate war damage. Among the respondents, 8174 (57.8%) were female and 5966 (42.2%) were male, with a mean (SD) age of 48.9 (16.8) years. Additionally, 5620 respondents (39.7%) had higher educational attainment, 12 544 (88.7%) were Ukrainian, 5648 (39.9%) were employed by others, and 1162 (8.2%) were self-employed. The data show that 2517 respondents (17.8%) experienced symptoms of mental disorder (ie, either feeling suicidal or helpless) after the war broke out.

**Figure 1.**
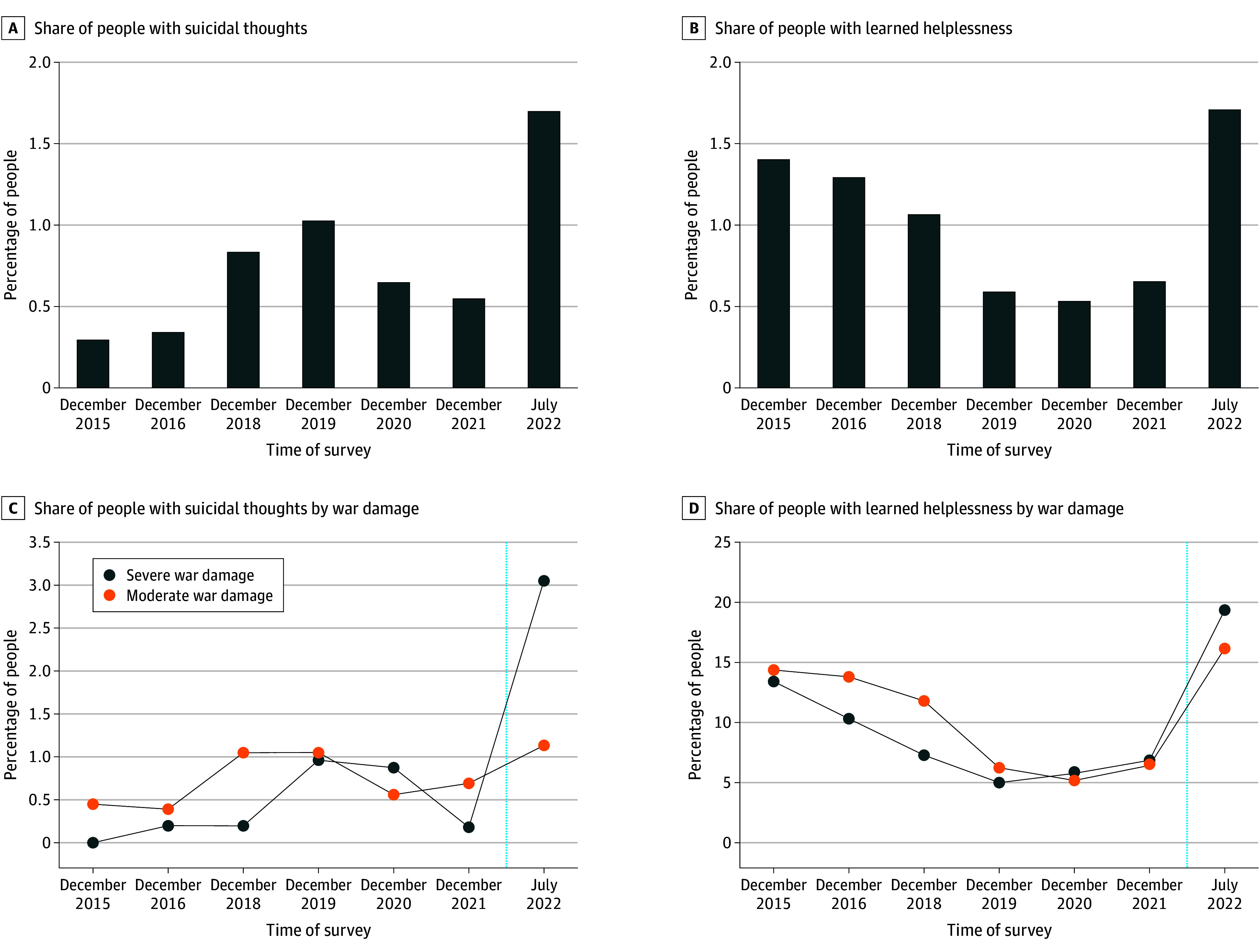
Suicidal Thoughts and Learned Helplessness Among Civilians in Ukraine Nationwide share of people with suicidal thoughts and learned helplessness by survey wave and by the extent of war damage (severe or moderate). The blue dotted line (parts C and D) represents the time when the war broke out (ie, February 2022).

Figure 1A shows that the share of people with suicidal thoughts increased dramatically after the outbreak of the war, rising from 0.55% in December 2021 to 1.70% in July 2022 (1.1 percentage points). This 2-fold increase was substantial even when compared with the increase in suicidal thoughts during 2017-2019 (ie, from 0.30% in December 2015, 0.34% in December 2016, 0.84% in December 2018, to 1.03% in December 2019), a period marked by several sociopolitical reforms,^38^ the dissolution of the much-hailed anticorruption campaign,^39^ and a major political change in Ukraine with the election of Volodymyr Zelensky as president.^40^ The share of people feeling helpless, as shown in Figure 1B, also increased nearly 3-fold after the breakout of the war, rising from 6.55% in December 2021 to 17.10% in July 2022.

**Figure 2.**
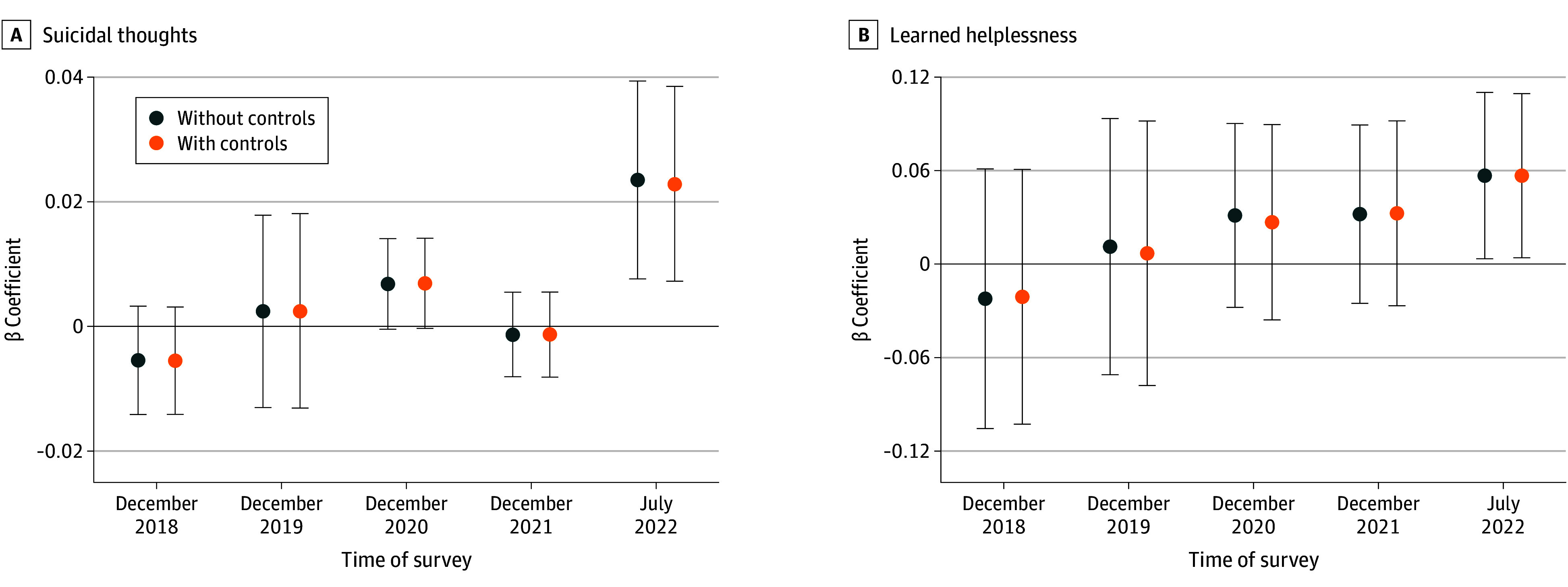
Interaction of War, Suicidal Thoughts and Learned Helplessness, Survey Wave, and War Damage in Ukraine The coefficients on the interaction term between the indicators of different survey waves (2018, 2019, 2020, 2021, and 2022) and the indicator measuring the extent (severe or moderate) of war damages from dynamic difference-in-differences regressions while controlling for oblast (province) fixed effects are shown. Control variables include sex, age, age-squared, educational level, nationality, and occupation. SEs are clustered at the oblast level. The error bars show 95% CIs. The outcome variables are suicidal thoughts and learned helplessness.

Figure 1C and D show that respondents from the 2 groups of oblasts with mild and severe war damage had similar levels of suicidal thoughts and helplessness from 2015 to 2021. Furthermore, an event-study analysis in Figure 2 and eTable 4 in Supplement 1 showed that before the war broke out, there were no significant differences in the mental health measures between the 2 groups of oblasts. Therefore, the parallel trend assumption holds for civilians’ mental health status before the war between the treated and control groups.

Further, in a more restrictive specification following model 1, Table 1 showed that the likelihood of civilians reportedly having thoughts of suicide after the breakout of the war was 2.2 percentage points (β estimate, 0.022 [SE, 0.006]; 95% CI, 0.009-0.036) higher in places that experienced severe war damage compared with areas with moderate war damage, and feelings of helplessness were 4.9 percentage points (β estimate, 0.049 [SE, 0.019]; 95% CI, 0.009-0.088) higher. These estimates were economically meaningful, representing 275% (0.022/0.008) and 47.1% (0.049/0.104) of the sample mean, respectively. The results were consistent with the pattern shown in Figure 1C and D. Given the absence of statistics that directly match suicidal thoughts and helplessness, we used disability-adjusted life years (DALYs) for major depressive disorder and dysthymia as proxies, respectively. While mindful of the risk of overextrapolation, our estimated increase in suicidality, using the 2019 statistics as a benchmark,^11^ translates to an estimated 42 million additional DALYs. Similarly, the surge in feelings of helplessness is estimated to correspond to nearly 17 million more DALYs.

**Table 1.** Difference-in-Differences Results Identifying the Association of War With Civilians’ Mental Health^a^

Model by dependent variable (N = 14 140)	β Estimate (SE) [95% CI]	*P* value
**Suicidal thoughts (mean: 0.008)**		
Model A: without controls, oblast FE, and survey FE, damage × post	0.022 (0.006) [0.009 to 0.036]	.002
Model B: without controls and with oblast FE and survey FE, damage × post	0.023 (0.006) [0.010 to 0.036]	.002
Model C: with controls, oblast FE, and survey FE, damage × post	0.022 (0.006) [0.009 to 0.036]	.002
**Learned helplessness (mean: 0.104)**		
Model A: without controls, oblast FE, and survey FE, damage × post	0.045 (0.021) [0.001 to 0.089]	.046
Model B: without controls and with oblast FE and survey FE, damage × post	0.048 (0.020) [0.007 to 0.089]	.03
Model C: with controls, oblast FE, and survey FE, damage ×	0.049 (0.019) [0.009 to 0.088]	.02

Abbreviations: FE, fixed effect; post, postwar survey time.

^a^ Mental health variables (suicidal thoughts and learned helplessness) were regressed on the interaction term between the indicator for the postwar period and an indicator for the extent of war damage (severe or moderate), while controlling for a range of individual characteristics, as well as oblast and survey FEs, in model C using model 1. Individual characteristics include sex, age, age-squared, educational level, nationality, and occupation. Variable definitions are provided in eTable 1 in Supplement 1. SEs clustered at the oblast level are reported in parentheses.

The results from the robustness checks, in which we changed the definition of war damage, are presented in eTable 5 in Supplement 1, and they are consistent with our baseline findings. Results shown in eTable 6 in Supplement 1 alleviate the stable unit treatment value of assumption concern further. For a sharper comparison between the most severely affected and the most moderately affected civilian counterparts, eTable 7 in Supplement 1 suggests that the share of respondents reportedly having suicidal thoughts increased by 2.6 percentage points (β estimate, 0.026 [SE, 0.007]; 95% CI, 0.009-0.043). Similarly, the fraction of people feeling helpless increased by 6.8 percentage points (β estimate, 0.068 [SE, 0.028]; 95% CI, 0.002-0.134). Furthermore, these findings were robust to changes in control variables, as shown in eTable 8 in Supplement 1.

### Heterogenous Association of War With Mental Health by Sex, Wealth, and Education

Table 2 summarizes the subsample findings using model 1. The β estimates suggested that the negative association between war exposure and mental health was mainly attributable to the high-wealth subpopulation. The estimates in this group were larger, showing increases of 5.6 percentage points (β estimate, 0.056 [SE, 0.021]; 95% CI, 0.012-0.100) in suicidal thoughts and 11.1 percentage points (β estimate, 0.111 [SE, 0.046]; 95% CI, 0.016-0.205) in helplessness compared with the baseline results of 2.2 and 4.9 percentage points, respectively.

**Table 2.** Heterogeneous Associations Between War Damage and Civilians’ Mental Health^a^

Dependent variable	Suicidal thoughts		Learned helplessness	
	β Estimate (SE) [95% CI]	*P* value	β Estimate (SE) [95% CI]	*P* value
Financial situation				
Low	0.019 (0.010) [−0.001 to 0.039]	.06	0.049 (0.024) [−0.0002 to 0.098]	.05
Middle	0.008 (0.007) [−0.005 to 0.022]	.21	0.008 (0.041) [−0.077 to 0.093]	.85
High	0.056 (0.021) [0.012 to 0.100]	.01	0.111 (0.046) [0.016 to 0.205]	.02
Sex				
Female	0.015 (0.005) [0.005 to 0.025]	.005	0.063 (0.024) [0.013 to 0.113]	.02
Male	0.030 (0.013) [0.004 to 0.056]	.02	0.032 (0.042) [−0.055 to 0.118]	.46
Financial situation and sex				
Low-female	0.015 (0.008) [−0.001 to 0.031]	.06	0.069 (0.031) [0.006 to 0.132]	.03
Low-male	0.023 (0.018) [−0.013 to 0.059]	.20	0.022 (0.050) [−0.081 to 0.125]	.66
Middle-female	0.008 (0.011) [−0.014 to 0.030]	.47	−0.003 (0.046) [−0.097 to 0.091]	.95
Middle-male	0.010 (0.010) [−0.011 to 0.032]	.34	0.008 (0.061) [−0.118 to 0.134]	.89
High-female	0.036 (0.031) [−0.027 to 0.100]	.25	0.244 (0.078) [0.082 to 0.406]	.005
High-male	0.066 (0.031) [0.001 to 0.130]	.046	0.036 (0.052) [−0.071 to 0.143]	.49
Educational level				
Primary or secondary	0.015 (0.011) [−0.008 to 0.038]	.19	0.043 (0.019) [0.003 to 0.083]	.04
Higher	0.033 (0.013) [0.005 to 0.060]	.02	0.053 (0.026) [−0.001 to 0.107]	.05
Age, y				
18-49 y	0.032 (0.008) [0.015 to 0.048]	<.001	0.050 (0.019) [0.011 to 0.090]	.02
50-95 y	0.012 (0.006) [−0.000 to 0.024]	.06	0.044 (0.027) [−0.012 to 0.099]	.12

^a^ Estimated coefficients of damage × post (postwar survey time) when exploring the heterogeneous association of war damage with individuals’ mental health using model 1 through subsample analyses, categorized by financial situation, sex, financial situation × sex, and educational level. The dependent variables include suicidal thoughts and learned helplessness. SEs clustered at the oblast level are reported in parentheses.

Further, although both sexes appeared to experience the adverse mental health impacts of the war, male respondents seemed more vulnerable to suicidal thoughts than woman, with an increase of 3.0 percentage points (β estimate, 0.030 [SE, 0.013]; 95% CI, 0.004-0.056) for men vs 1.5 percentage points (β estimate, 0.015 [SE, 0.005]; 95% CI, 0.005 to 0.025) for women, while female respondents were more at risk of feeling helpless, with an increase of 6.3 percentage points (β estimate, 0.063 [SE, 0.024]; 95% CI, 0.013-0.113) vs 3.2 percentage points (β estimate, 0.032 [SE, 0.042]; 95% CI, −0.055 to 0.118) for men. The increases were primarily attributable to wealthy male respondents for suicidal thoughts, while the likelihood of feeling helpless after the war was largely attributable to wealthy female respondents.

Our analysis of the varying impacts of war on different educational levels revealed that more educated individuals exhibit a stronger psychological response to the conflict. Specifically, the likelihood of having suicidal thoughts increased by 3.3 percentage points (β estimate, 0.033 [SE, 0.013]; 95% CI, 0.005-0.060), and feelings of helplessness increased by 5.3 percentage points (β estimate, 0.053 [SE, 0.026]; 95% CI, −0.001 to 0.107) among more educated respondents following the start of the war. Additionally, respondents 49 years or younger were more sensitive to the war in terms of suicidal thoughts (β estimate, 0.032 [SE, 0.008]; 95% CI, 0.015-0.048) and feelings of helplessness (β estimate, 0.050 [SE, 0.019]; 95% CI, 0.011-0.090). The results are robust to incorporating the interaction terms of control variables and indicators for war damage and after war period (details shown in eTable 9 in Supplement 1).

### Mechanisms Underlying Associations Between War and Mental Health

Further, following model (1) in the main text and using 4 mechanism variables, including loss of jobs, experience of bombings, living without a livelihood, and loss of loved ones, as dependent variables, the DID results shown in eTable 10 in Supplement 1 confirmed the empirical patterns observed in the raw data shown in eFigure 1 in Supplement 1. The share of job losses was 9.7 percentage points (β estimate, 0.097 [SE, 0.032]; 95% CI, 0.032-0.162) higher in severely damaged areas compared to moderately damaged ones after the outbreak of the war, representing over 100% (0.097/0.097) increases relative to the sample average. There were additional increases of 29.6 percentage points (β estimate, 0.296 [SE, 0.081]; 95% CI, 0.128-0.463) in experience of bombing and 4.0 percentage points (β estimate, 0.040 [SE, 0.016]; 95% CI, 0.007-0.074), in experiencing and living without a livelihood for people in more severely damaged places relative to others (details are shown in eAppendix 3 in Supplement 1) .

### Immediate Mental Health Outcomes After Bombing

eFigure 2 in Supplement 1 shows that following the attack, the share of people having suicidal thoughts increased by 2.7 (95% CI, 4.0-1.3) percentage points; those losing faith in themselves, by 4.4 (95% CI, 8.3-3.9) percentage points; and those feeling helpless, by 5.4 (95% CI, 24.3-18.9) percentage points. The balance tests represented in eFigure 2 and eTable 11 in Supplement 1 suggested that people interviewed before the bombing were statistically similar to those interviewed afterward across a wide range of characteristics. Then, eFigure 2 and eTable 12 in Supplement 1 presented the results using model A1. The estimates suggested that the probabilities increased by 2.8 percentage points (β estimate, 0.028 [SE, 0.014]; 95% CI, 0.001-0.055) for having suicidal thoughts, by 5.0 percentage points (β estimate, 0.050 [SE, 0.020]; 95% CI, 0.010-0.090) for losing faith in themselves, and by 5.8 percentage points (β estimate, 0.058 [SE, 0.035]; 95% CI, −0.010 to 0.126) for feeling helpless (detailed description is found in eAppendix 4 in Supplement 1).

## Discussion

In this cohort study, we found that the ongoing Russia-Ukraine conflict was associated with an increase in severe mental distress among civilians in Ukraine. As a point of reference, recent research studying changes in mental health conditions before and after the 2014 Russia-Ukraine war found that 50% of Ukrainians exhibited stress symptoms in 2016 compared with 45% in 2012—a much milder increase relative to our estimates.^41^ However, given the difference in scale and impact between the 2014 and 2022 wars, our findings are reasonable. Further, our estimate of 355 of 2000 Ukrainians (17.8%) experiencing symptoms of mental disorder (ie, either feeling suicidal or helpless) after war is largely consistent with cross-sectional studies examining the postwar prevalence of mental distress.^42,43^ If anything, our findings are on the conservative side.^11,44^ As the war progresses, experts believe that the mental health status of Ukrainians will further deteriorate.^45^ Our analysis on the association between war and mental health in the short term, presented in eAppendix 4 in Supplement 1 (due to limited space here), revealed a qualitatively similar pattern. This study contributes to the literature by providing novel evidence on the association between exposure to war and the mental health of the general public, complementing the existing studies that rely solely on postevent surveys with cross-sectional correlations or focus on specific population groups.^15,36^

The heterogeneous results showed that wealthy people experienced more mental health adversity from the war. While they have more resources to withstand the negative impact of war, they also have more to lose compared with their less affluent peers. Moreover, men were more likely to have suicidal thoughts, and women were more likely to feel helpless. These findings align with the insight that men tend to value independence and decisiveness and may regard acknowledging the need for help as a weakness, whereas women typically view seeking help as neutral.^46^ Research by Ditlevsen and Elklit^47^ also emphasizes that men tend to internalize stress, potentially manifesting as suicidal ideation, while women are more likely to experience affective symptoms such as helplessness and depression due to their heightened emotional processing. From an economic perspective, studies such as that of Autor et al^48^ show that men, particularly those in economically strained regions, are disproportionately impacted by adverse labor market shocks, which may compound feelings of despair and suicidal tendencies. Similarly, Li and Wang^49^ found that women often bear a disproportionate burden of caregiving and emotional labor during crises, which can exacerbate feelings of helplessness. Therefore, when faced with severe damage, men are less likely to feel helpless but more likely to have suicidal thoughts directly, while women exhibit the opposite pattern.

As for the potential explanations regarding why people with higher levels of education are more vulnerable to increased likelihood of suicidal thoughts and feelings of helplessness, on the one hand, they may have more intangible assets to help them cope with the war; on the other hand, they also have more to lose when their country is at war. In the present study, it seems that the second explanation dominates the first.

The channel analysis revealed that job loss, exposure to bombings, and difficulties in sustaining a livelihood were the primary mechanisms underlying the association between exposure to war and mental distress in our sample. The results confirmed previous observations that the war has led to the closure of hundreds of businesses and that the impact of war on employment can be devastating.^25^ The lack of employment opportunities may have imposed important mental stress, as people rely on their jobs to sustain their livelihoods. Meanwhile, the fear and trauma caused by ongoing and sudden attacks can leave people frightened and vulnerable. Additionally, the destruction of residential buildings and infrastructure, and the consequent shortages of water and electricity, have made it difficult for civilians to sustain their livelihoods.

### Limitations

We acknowledge several limitations in our study. First, our sample was limited to individuals living in Ukraine during the war, excluding refugees who fled to other countries. This could lead to an underestimation or overestimation of the associations, as we do not have data on refugees. For one thing, refugees may have higher socioeconomic status since they are more likely to have the resources to leave the country. Combining the heterogeneous finding that wealthy and highly educated respondents tend to experience more mental disorders, this suggests an underestimation. On the other hand, if respondents chose to stay in Ukraine rather than leave, they may feel more desperate about the future. As a result, the average mental disorder status of respondents could be worse than that of refugees, leading to overestimation. Second, the intervals between the 7 survey waves were not uniform, which could introduce white noise when constructing mental health dummy variables. Third, while our mental health measures are supported by existing literature, a more detailed assessment of civilians’ mental disorders could potentially provide a clearer picture.

## Conclusions

Using a survey dataset and 2 identification strategies in this cohort study, we quantified the association between mental health and exposure to the Russia-Ukraine war. We found that following the outbreak of the war, the share of people reporting living in a state close to suicide increased by 1.1 percentage points (from 0.6% to 1.7%), nearly tripling relative to the prewar level. The share of people feeling helpless also nearly tripled, rising from 6.6% in December 2021 to 17.1% in July 2022. We found that the association operated through loss of employment and intermittent bombings. These findings suggest that policymakers should prioritize creating job opportunities and ensuring a safe living environment to support citizens’ recovery in the aftermath of conflict.

## Notes

Supplement 1. **eAppendix 1.** Background of Russia-Ukraine Conflict **eAppendix 2.** Empirical Strategy to Investigate the Short-Term Association Between War and Civilians’ Mental Health **eAppendix 3.** Mechanisms Linking War to Mental Health **eAppendix 4.** The Immediate Mental Health Impact of Bombing **eFigure 1.** War and Job Loss, Bombing Experience, Being Without a Livelihood, and Death of Loved Ones **eFigure 2.** War and Mental Health Conditions in the Short Term **eTable 1.** Variable Definition **eTable 2.** Summary Statistics: Full Sample **eTable 3.** Summary Statistics: The July 2022 Wave **eTable 4.** Tests on Parallel Trend **eTable 5.** War and Civilians’ Mental Health: Alternative Thresholds When Defining War Damages **eTable 6.** War and Civilians’ Mental Health: Stable Unit Treatment Value of Assumption (SUTVA) Test **eTable 7.** War and Civilians’ Mental Health: Alternative Definition of War Intensity **eTable 8.** War and Civilians’ Mental Health: Change Control Variables **eTable 9.** Robustness Tests on Heterogeneous Impacts **eTable 10.** Linking War to Mental Health Conditions **eTable 11.** Balanced Tests for Short-Term Analyses **eTable 12.** War and Civilians’ Mental Health in the Short Term

Supplement 2.
Data Sharing Statement
