# Supplementary material for: Mental Health of Residents of Ukraine Exposed to the Russia-Ukraine Conflict
Source: JAMA Netw Open. 2025 Feb 13;8(2):e2459318. doi: 10.1001/jamanetworkopen.2024.59318 (PMC11826354; doi:10.1001/jamanetworkopen.2024.59318)
Supplement: Supplement 1. — eAppendix 1. Background of Russia-Ukraine Conflict eAppendix 2. Empirical Strategy to Investigate the Short-Term Association Between War and Civilians’ Mental Health eAppendix 3. Mechanisms Linking War to Mental Health eAppendix 4. The Immediate Mental Health Impact of Bombing eFigure 1. War and Job Loss, Bombing Experience, Being Without a Livelihood, and Death of Loved Ones eFigure 2. War and Mental Health Conditions in the Short Term eTable 1. Variable Definition eTable 2. Summary Statistics: Full Sample eTable 3. Summary Statistics: The July 2022 Wave eTable 4. Tests on Parallel Trend eTable 5. War and Civilians’ Mental Health: Alternative Thresholds When Defining War Damages eTable 6. War and Civilians’ Mental Health: Stable Unit Treatment Value of Assumption (SUTVA) Test eTable 7. War and Civilians’ Mental Health: Alternative Definition of War Intensity eTable 8. War and Civilians’ Mental Health: Change Control Variables eTable 9. Robustness Tests on Heterogeneous Impacts eTable 10. Linking War to Mental Health Conditions eTable 11. Balanced Tests for Short-Term Analyses eTable 12. War and Civilians’ Mental Health in the Short Term [file jamanetwopen-e2459318-s001.pdf]

Supplementary Online Content

An J, Wang T, Chen B, Oleksiyenko A, Lin C. Mental health of residents of Ukraine exposed to the Russia-Ukraine conflict. *JAMA Netw Open*. 2025;8(2):e2459318. doi:10.1001/jamanetworkopen.2024.59318

eAppendix 1. Background of Russia-Ukraine Conflict

eAppendix 2. Empirical Strategy to Investigate the Short-Term Association Between War and Civilians' Mental Health

eAppendix 3. Mechanisms Linking War to Mental Health

eAppendix 4. The Immediate Mental Health Impact of Bombing

eFigure 1. War and Job Loss, Bombing Experience, Being Without a Livelihood, and Death of Loved Ones

eFigure 2. War and Mental Health Conditions in the Short Term

eTable 1. Variable Definition

eTable 2. Summary Statistics: Full Sample

eTable 3. Summary Statistics: The July 2022 Wave

eTable 4. Tests on Parallel Trend

eTable 5. War and Civilians' Mental Health: Alternative Thresholds When Defining War Damages

eTable 6. War and Civilians' Mental Health: Stable Unit Treatment Value of Assumption (SUTVA) Test

eTable 7. War and Civilians' Mental Health: Alternative Definition of War Intensity

eTable 8. War and Civilians' Mental Health: Change Control Variables

eTable 9. Robustness Tests on Heterogeneous Impacts

eTable 10. Linking War to Mental Health Conditions

eTable 11. Balanced Tests for Short-Term Analyses

eTable 12. War and Civilians' Mental Health in the Short Term

This supplementary material has been provided by the authors to give readers additional information about their work.

© 2025 An J et al. *JAMA Network Open*.

eAppendix 1. Background of Russia-Ukraine Conflict

In this section, we detail the socio-economic and political information of Ukraine, and the evolution of conflicts between Russia and Ukraine.

Ukraine, located in Eastern Europe, is the second-largest country on the continent by area, surpassed only by Russia. It has a population of approximately 41 million people, with Kyiv as its capital and largest city. Ukraine gained independence from the Soviet Union in 1991, marking the start of its journey as a sovereign state.

Economically, Ukraine is endowed with abundant natural resources, including fertile agricultural land ideal for grain production, earning it the nickname “breadbasket of Europe.” The country also boasts significant coal and mineral deposits and a well-developed industrial base, spanning sectors such as machinery, aerospace, chemicals, and metallurgy. Despite these assets, Ukraine has faced persistent economic challenges, including corruption, inefficiency in state-owned enterprises, and the need for extensive structural reforms. As a result, its GDP per capita remains relatively low compared to other European nations.

Politically, Ukraine has undergone substantial turmoil and transformation since its independence. The nation has oscillated between pro-European and pro-Russian orientations, reflecting a divided populace. Notable political events include the 2004 Orange Revolution, a series of protests and political upheaval following a disputed presidential election, and the 2013–2014 Euromaidan protests. The latter led to the ousting of President Viktor Yanukovych after his decision to suspend an association agreement with the European Union in favor of closer ties with Russia.

The conflict between Russia and Ukraine has deep historical roots but escalated significantly in the 21st century. The most pivotal phase began in 2014 with Russia’s annexation of Crimea, a strategic peninsula in the Black Sea, following the Euromaidan protests and Yanukovych’s removal from office. Subsequently, pro-Russian separatists in the eastern regions of Donetsk and Luhansk declared independence, triggering armed conflict with Ukrainian government forces. Despite several ceasefire agreements, including the Minsk Protocols, the conflict has continued with periodic escalations.

This geopolitical struggle has been characterized by economic sanctions imposed on Russia by Western nations, strong support for Ukraine’s sovereignty from the European Union and the United States, and a complex landscape of information warfare. Ukraine remains committed to closer integration with the West, aspiring to European Union membership and NATO partnership while navigating its fraught relationship with Russia.

© 2025 An J et al. *JAMA Network Open*.

On February 24, 2022, Russian President Vladimir Putin announced a "special military operation" aimed at the "demilitarization and denazification" of Ukraine, asserting that Russia had no intention of occupying the country. Fighting began at 3:40 a.m. Kyiv time in Luhansk Oblast, near Milove on the border with Russia. Russian forces launched major infantry and tank assaults in four spearheads: a northern front from Belarus targeting Kyiv, a southern front from Crimea, a southeastern front from Russian-controlled Donbas, and an eastern front from Russia towards Kharkiv and Sumy. As of early 2023, when this article was written, the conflict remains ongoing.

© 2025 An J et al. *JAMA Network Open*.

eAppendix 2. Empirical Strategy to Investigate the Short-Term Association Between War and Civilians' Mental Health

We also investigated the association between Russia's bombing and people's mental health in the very short-term using an alternative strategy. During the period from July 6 to July 18, 2022, several survey locations were bombed. This created random exposure to the bombing for people interviewed before and after the event, allowing us to study the short-term relationship between the war and mental health by comparing the mental health status of respondents interviewed immediately before and after a wave of intensive bombings within the same locality (i.e., settlement, which is a subordinate locality unit within an oblast), while controlling for other confounding factors. We used an OLS model in Stata as follows:

$$MH_{ijt} = \beta After_{it} + \alpha_1 Controls_{ijt} + FEs + \varepsilon_{ijt} \qquad \text{(A1)}$$

where i, j, t denote individual, settlement and interview date, respectively. MH_{ijt} represents one of the mental health measures: *Suicidal*, *Helpless* or *Lose faith in themselves*. $After_{it}$is a dummy variable that equals 1 if the individual's interview date is after the July 12 attack, and zero otherwise. $Control_{ijt}$ is the same as in model (1) in the main text. We further controlled settlement fixed effect.

© 2025 An J et al. *JAMA Network Open*.

eAppendix 3. Mechanisms Linking War to Mental Health

Theoretically, losing jobs, experiencing bombings, living without a livelihood, and losing loved ones can potentially harm one's mental health. From the raw data shown in eFigure 1 in Supplement 1 here, we found that, five months into the war, the shares of people who lost their jobs, experienced bombing, and lived without a livelihood in severely-damaged places were much higher than that in areas with moderate damages.

[Insert eTable 11 Here]

Further, following model (1) in the main text and using four mechanism variables as dependent variables, the DID results shown in eTable 11 here, Panel A confirmed the empirical patterns observed in the raw data. The share of job losses was 9.7 pp (β [SE], 0.097 [0.032]; 95% CI, 0.032 to 0.162) higher in severely damaged areas compared to moderately damaged ones after the outbreak of the war, representing over a 100% (0.097/0.097) increases relative to the sample average. There were additional 29.6 pp (β [SE], 0.296 [0.081]; 95% CI, 0.128 to 0.463) and 4.0 pp (β [SE], 0.040 [0.016]; 95% CI, 0.007 to 0.074) increases, respectively, in *Experiencing bombing* and *Without a livelihood* for people in more severely damaged places relative to others.

While it is true that approximately 7,000 civilians have been killed and 11,000 more injured as a result of the war, column (4) revealed that the negative impact of war damage on mental health cannot be solely attributed to the increasing share of respondents who have experienced the death of loved ones, as the interaction term was not significant. When we added the mechanism variable to the baseline model, where mental conditions are dependent variables, the results shown in eTable 11, Panels B and C indicate lower estimated magnitude of *Damage × Post*, except for small decreases in regressions controlling for *Death of loved ones*, consistent with the channels we discussed above.

© 2025 An J et al. *JAMA Network Open*.

eAppendix 4. The Immediate Mental Health Impact of Bombing

Lastly, we investigated the very short-term impact of Russia's bombing using the July 2022 wave of the KIIS survey. eFigure 2(a) here showed that following the attack, the share of people having suicidal thoughts, losing faith in themselves, and feeling helpless increased by 2.7 pp (4.0%-1.3%), 4.4 pp (8.3%-3.9%), and 5.4 pp (24.3%-18.9%), respectively.

[Insert eFigure 2 Here]

For our results to be convincing, it is crucial that the individuals interviewed by KIIS before and after the bombings are similar, except for their exposure to the bombings. The balance tests represented in eFigure 2(b) and eTable 6 in Supplement 1 here suggested that people interviewed before the bombing were statistically similar to those interviewed afterward across a wide range of characteristics.

Then, eFigure 2(c) and eTable 7 in Supplement 1 presented the results using model (A1). The estimates suggested that the probabilities increased by 2.8 pp (β [SE], 0.028 [0.014]; 95% CI, 0.001 to 0.055), 5.8 pp (β [SE], 0.058 [0.035]; 95% CI, -0.010 to 0.126) and 5.0 pp (β [SE], 0.050 [0.020]; 95% CI, 0.010 to 0.090) for *Suicidal*, *Helpless*, and *Lose faith in themselves*, respectively. Regardless of how the standard errors were clustered—using robust standard errors, standard errors clustered at the settlement level, or bootstrapped standard errors at the settlement-level—the coefficients for *After* were all statistically significant at the conventional significance levels.

© 2025 An J et al. *JAMA Network Open*.

eFigure 1. War and Job Loss, Bombing Experience, Being Without a Livelihood, and Death of Loved Ones

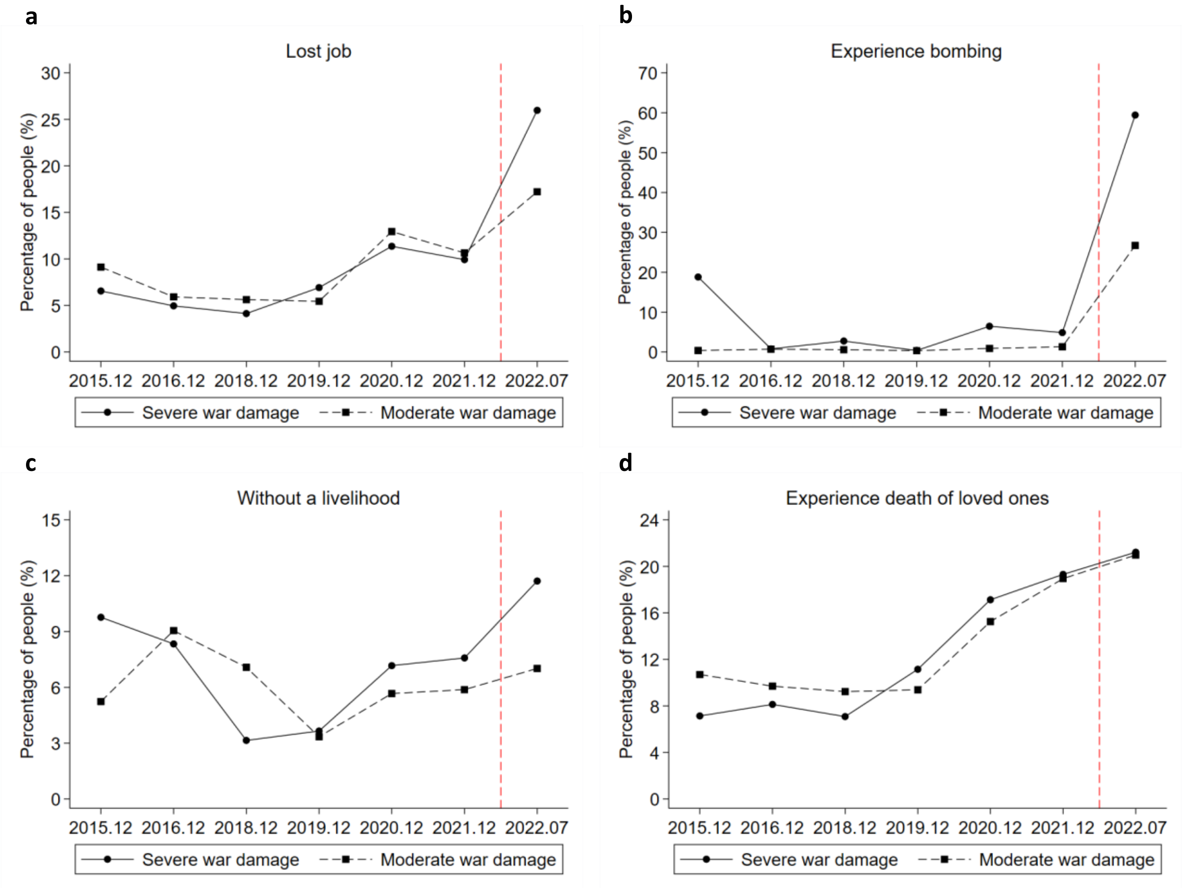

Notes: This figure plots the share of people ever lost jobs, experienced bombing or shelling, lived without a livelihood, and experienced the death of loved ones from 2015 to 2022 by the extent of their exposure to war damages. The red dashed line represents the moment when the war broke out. The horizontal axis is the time when the surveys were conducted.

© 2025 An J et al. *JAMA Network Open*.

eFigure 2. War and Mental Health Conditions in the Short Term

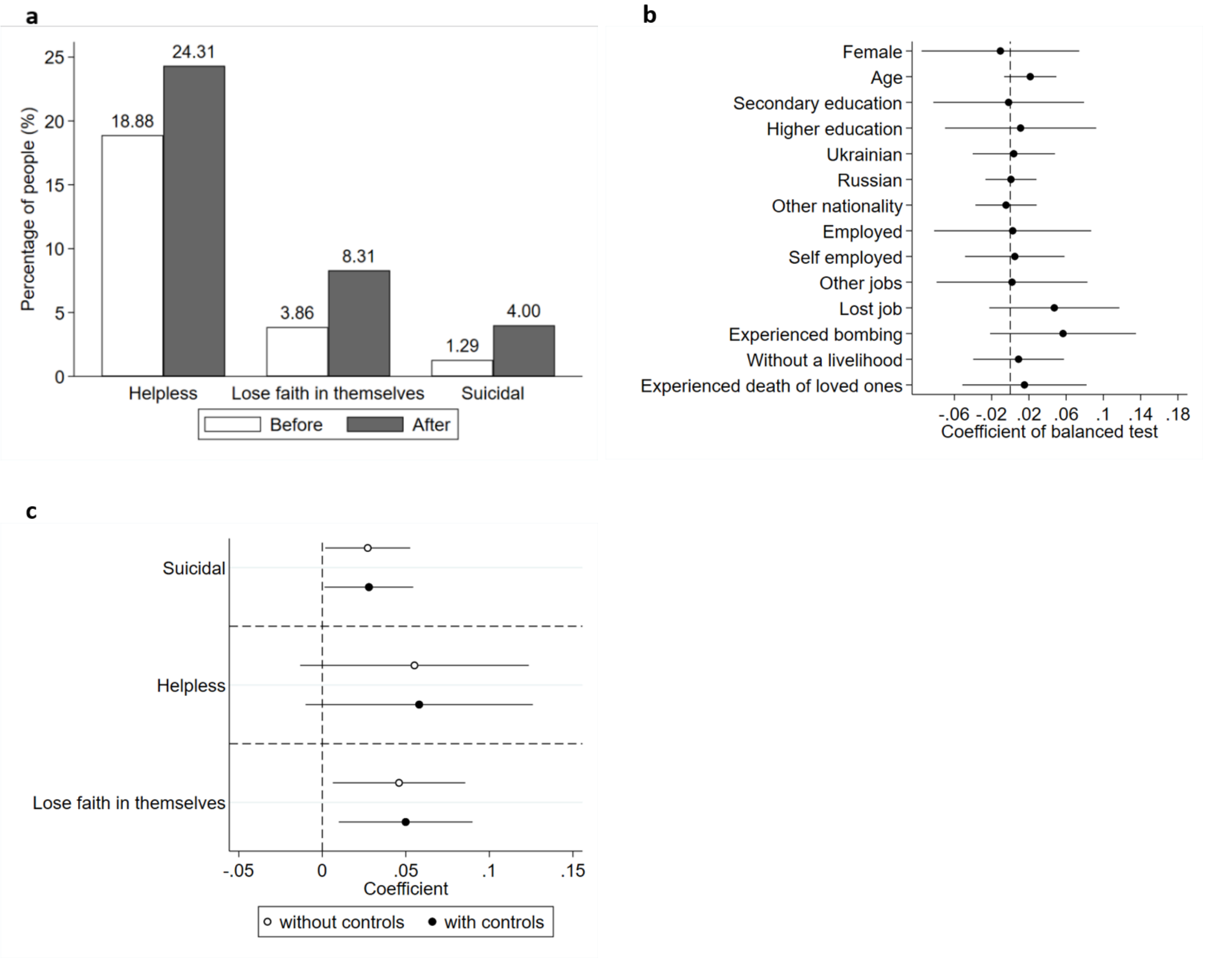

Notes: Panel (a) plots the share of people with suicidal thoughts, learned helplessness, and those who lost faith in themselves before (from July 6 to July 12) and after (from July 13 to July 18) the Russian bombings. Panel (b) visualizes the results from the balance tests on the two groups of respondents interviewed just before and after the Russian bombings across various demographic characteristics and experiences. Panel (c) shows the coefficients of the post-bombing indicator when we regress mental health variables on the post-bombing period indicator, while controlling for settlement fixed effects and employing robust standard errors. Controls include respondents' gender, age, age squared, education, nationality, and occupation. The error bars represent 95% confidence intervals.

© 2025 An J et al. *JAMA Network Open*.

eTable 1. Variable Definition

Variables	Definition
Suicidal	A dummy variable equals to 1 if the respondent has lived in a state close to suicide during the year, and 0 otherwise.
Helpless	A dummy variable equals to 1 if the respondent has felt helpless during the year, and 0 otherwise.
Damage	A dummy variable equals to 1 if the oblast has war damages over $5 billion by June 1, 2022, including Donetska, Luhanska, Kharkivska, Kyivska, Chernihivska and Zaporizka, and 0 for other oblasts.
$Damage_a$	A dummy variable equals to 1 if the oblast has war damages over $10 billion by June 1, 2022, including Donetska, Luhanska, Kharkivska and Kyivska, and 0 for Kirovohradska, Cherkaska, Poltavska and Dnipropetrovska.
Damage75	A dummy variable equals to 1 if the oblast experienced at least $7.5 billion in losses and 0 otherwise.
Damage1	A dummy variable indicating whether the oblast’s damage exceeds $1 billion.
D.avg.attacks	A dummy variable equals to 1 if the number of military attacks is higher than the average level across the oblasts, and 0 otherwise.
D.median.attacks	A dummy variable equals to 1 if the number of military attacks is higher than the median level across the oblasts, and 0 otherwise.
$Damage_r$	A dummy variable equals to 1 if the oblast experiences damage loss higher than $5 billion, including six oblasts: Donetska, Luhanska, Kharkivska, Kyivska, Zaporizka, and Chernihivska, and 0 if the oblast experienced the least damage (war damage between $0 and $0.2 billion), including Chernivtsi, Volynska, Ternopilska, Ivano-Frankivsk, Khmelnytsky, and Rivne.
Post	A dummy variable equals to 1 if the interview date is after February 24, 2022, and 0 otherwise.
After	A dummy variable equals to 1 if the interview date is after July 12, 2022 for the July 2022 wave survey, and 0 otherwise.
Female	A dummy variable equals to 1 if the respondent is female, and 0 otherwise.
Age	Respondent’s age.
Primary education	A dummy variable equals to 1 if the respondent's education level is less or equal to primary education, and 0 otherwise.
Secondary education	A dummy variable equals to 1 if the respondent has education level that is above primary but below or equal to secondary education, and 0 otherwise.
Higher education	A dummy variable equals to 1 if the respondent's education level is above secondary, and 0 otherwise.

© 2025 An J et al. *JAMA Network Open*.

Ukrainian	A dummy variable equals to 1 if the respondent considers himself or herself as a Ukrainian, and 0 otherwise.
Russian	A dummy variable equals to 1 if the respondent considers himself or herself as a Russian, and 0 otherwise.
Other nationality	A dummy variable equals to 1 if the respondent identifies himself or herself as a national from Belarus, Moldovan, Crimean Tatar, Bulgarian, Hungarian, Romanians, Pole, Jew and others, and 0 otherwise.
Employed	A dummy variable equals to 1 if the respondent is employed as an agricultural worker, an employee, a specialist, or a soldier, and 0 otherwise.
Self employed	A dummy variable equals to 1 if respondent is engaged in respondent work, entrepreneur, owner of his own business, or farmer, and 0 otherwise.
Other jobs	A dummy variable equals to 1 if the respondent is a student, unemployed, retired, running a household or in other occupations, and 0 otherwise.
Lost job	A dummy variable equals to 1 if the respondent has lost his/her job and been forced into unemployment during the year, and 0 otherwise.
Low finance	A dummy variable equals to 1 if the respondent claimed that "We do not have enough money even for food", or "We have enough money for food, but buying clothes is already difficult".
Mid finance	A dummy variable equals to 1 if the respondent claimed that "We have enough money for food, clothes and we can save some, but this is not enough to buy expensive things (such as a refrigerator or TV)".
High finance	A dummy variable equals to 1 if the respondent claimed that "We can afford to buy some expensive things (such as a TV or fridge)", or "We can afford to buy whatever we want".
Experience bombing	A dummy variable equals to 1 if the respondent has experienced bombing or shelling during the year, and 0 otherwise.
Without a livelihood	A dummy variable equals to 1 if the respondent has been living without a livelihood during the year, and 0 otherwise.
Death of loved	A dummy variable equals to 1 if the respondent has experienced the death of loved ones during the year, and 0 otherwise.
Lose faith in themselves	A dummy variable equals to 1 if the respondent has lost faith in themselves during the year, and 0 otherwise.

© 2025 An J et al. *JAMA Network Open*.

eTable 2. Summary Statistics: Full Sample

Variables	Obs.	Mean	Median	Sd	P5	P10	P25	P75	P90	p95	Min	Max
Suicidal	14140	0.0077	0	0.0875	0	0	0	0	0	0	0	1
Helpless	14140	0.1037	0	0.3049	0	0	0	0	1	1	0	1
Damage	14140	0.2781	0	0.4481	0	0	0	1	1	1	0	1
$Damage_a$	5458	0.5227	1	0.4995	0	0	0	1	1	1	0	1
Post	14140	0.1414	0	0.3485	0	0	0	0	1	1	0	1
Female	14140	0.5781	1	0.4939	0	0	0	1	1	1	0	1
Age	14140	48.8641	49	16.8027	22	26	35	62	71	76	18	95
Primary education	14140	0.0087	0	0.0929	0	0	0	0	0	0	0	1
Secondary education	14140	0.5883	1	0.4922	0	0	0	1	1	1	0	1
Higher education	14140	0.3975	0	0.4894	0	0	0	1	1	1	0	1
Ukrainian	14140	0.8871	1	0.3164	0	0	1	1	1	1	0	1
Russian	14140	0.0569	0	0.2316	0	0	0	0	0	1	0	1
Other nationality	14140	0.0495	0	0.2169	0	0	0	0	0	0	0	1
Employed	14140	0.3994	0	0.4898	0	0	0	1	1	1	0	1
Self employed	14140	0.0822	0	0.2746	0	0	0	0	0	1	0	1
Other jobs	14140	0.5120	1	0.4999	0	0	0	1	1	1	0	1
Lost job	14140	0.0965	0	0.2952	0	0	0	0	0	1	0	1
Experience bombing	14140	0.0709	0	0.2566	0	0	0	0	0	1	0	1
Without a livelihood	14140	0.0657	0	0.2478	0	0	0	0	0	1	0	1
Death of loved	14140	0.1328	0	0.3394	0	0	0	0	1	1	0	1

Notes: This table presents the summary statistics for the full sample from 2015 to 2022. The full sample consists of 25 oblasts in Ukraine. Variable definitions are shown in eTable 1.

© 2025 An J et al. *JAMA Network Open*.

eTable 3. Summary Statistics: The July 2022 Wave

Variables	Obs.	Mean	Median	Sd	P5	P10	P25	P75	P90	p95	Min	Max
Suicidal	558	0.0287	0	0.1670	0	0	0	0	0	0	0	1
Helpless	558	0.2204	0	0.4149	0	0	0	0	1	1	0	1
Lose faith in themselves	558	0.0645	0	0.2459	0	0	0	0	0	1	0	1
After	558	0.5824	1	0.4936	0	0	0	1	1	1	0	1
Female	558	0.5358	1	0.4992	0	0	0	1	1	1	0	1
Age	558	46.0072	45	16.7376	21	25	32	60	70	74	18	93
Secondary education	558	0.3746	0	0.4844	0	0	0	1	1	1	0	1
Higher education	558	0.6219	1	0.4854	0	0	0	1	1	1	0	1
Ukrainian	558	0.9265	1	0.2612	0	1	1	1	1	1	0	1
Russian	558	0.0269	0	0.1619	0	0	0	0	0	0	0	1
Other nationality	558	0.0376	0	0.1905	0	0	0	0	0	0	0	1
Employed	558	0.5125	1	0.5003	0	0	0	1	1	1	0	1
Self employed	558	0.1147	0	0.3189	0	0	0	0	1	1	0	1
Other jobs	558	0.3692	0	0.4830	0	0	0	1	1	1	0	1
Lost job	558	0.2348	0	0.4242	0	0	0	0	1	1	0	1
Experience bombing	558	0.4857	0	0.5002	0	0	0	1	1	1	0	1
Without a livelihood	558	0.0914	0	0.2884	0	0	0	0	0	1	0	1
Death of loved	558	0.1953	0	0.3968	0	0	0	0	1	1	0	1

Notes: This table shows the summary statistics for the July 2022 wave survey (from July 6, 2022 to July 18, 2022). It consists of five settlements that meet two requirements: (1) more than 30 respondents were interviewed and (2) suffered Russian bombings during the thirteen-day interview window. Variable definitions are shown in eTable 1.

© 2025 An J et al. *JAMA Network Open*.

eTable 4. Tests on Parallel Trend

Dependent variable	Suicidal		Helpless	
	(1)	(2)	(3)	(4)
Damage × 2018	-0.0054	-0.0055	-0.0222	-0.0210
	(0.0042)	(0.0042)	(0.0404)	(0.0396)
Damage × 2019	0.0024	0.0025	0.0112	0.0069
	(0.0075)	(0.0076)	(0.0399)	(0.0411)
Damage × 2020	0.0068*	0.0069*	0.0312	0.0269
	(0.0035)	(0.0035)	(0.0286)	(0.0304)
Damage × 2021	-0.0013	-0.0013	0.0321	0.0326
	(0.0033)	(0.0033)	(0.0277)	(0.0288)
Damage × Post	0.0235***	0.0229***	0.0568**	0.0568**
	(0.0077)	(0.0076)	(0.0259)	(0.0255)
Controls	No	Yes	No	Yes
Oblast FE	Yes	Yes	Yes	Yes
Survey FE	Yes	Yes	Yes	Yes
Adj. R2	0.0041	0.0046	0.0283	0.0404
Observations	14140	14140	14140	14140

Notes: This table shows the results of testing the parallel trend assumption using an event-study approach by regressing the outcome variables—i.e., thoughts of suicide and learned helplessness—on the interactions between the 2018, 2019, 2020, 2021, and 2022 (Post) dummies and an indicator of the extent of war damage, while controlling for oblast and survey fixed effects. In columns (2) and (4), we further include control variables such as gender, age, age squared, education, nationality, and occupation. Variable definitions are provided in eTable 1. The abbreviation "FE" represents "Fixed Effects." Standard errors clustered at the oblast level are reported in parentheses. *, **, and *** indicate significance at the 10%, 5%, and 1% levels, respectively.

© 2025 An J et al. *JAMA Network Open*.

eTable 5. War and Civilians' Mental Health: Alternative Thresholds When Defining War Damages

Dep. Variable	Suicidal				Helpless			
	(1)	(2)	(3)	(4)	(5)	(6)	(7)	(8)
Damage75 × Post	0.0211***				0.0451*			
	(0.0072)				(0.0228)			
Damage1 × Post		0.0129*				0.0562***		
		(0.0070)				(0.0158)		
D.avg.attacks × Post			0.0154**				0.0622***	
			(0.0061)				(0.0143)	
D.median.attacks × Post				0.0160**				0.0660***
				(0.0059)				(0.0137)
Controls	Yes	Yes	Yes	Yes	Yes	Yes	Yes	Yes
Oblast FE	Yes	Yes	Yes	Yes	Yes	Yes	Yes	Yes
Survey FE	Yes	Yes	Yes	Yes	Yes	Yes	Yes	Yes
Adj. R2	0.0042	0.0037	0.0039	0.0040	0.0400	0.0406	0.0407	0.0408
Observations	14140	14140	14140	14140	14140	14140	14140	14140

Notes: This table presents the results from the robustness tests using an alternative threshold to define whether an oblast suffers from severe or moderate war damage, following model (1). Damage75 equals 1 if the oblast experienced at least $7.5 billion in losses and 0 otherwise. Damage1 is a dummy variable indicating whether the oblast's damage exceeds $1 billion. D.avg.attacks and D.median.attacks are dummy variables that equal 1 if the number of military attacks is higher than the average or median level across the oblasts, and 0 otherwise. Control variables include gender, age, age squared, education, nationality, and occupation. Variable definitions are provided in eTable 1. The abbreviation "FE" represents "Fixed Effects." Standard errors clustered at the oblast level are reported in parentheses. *, **, and *** indicate significance at the 10%, 5%, and 1% levels, respectively.

© 2025 An J et al. *JAMA Network Open*.

eTable 6. War and Civilians' Mental Health: Stable Unit Treatment Value of Assumption (SUTVA) Test

Dep. variable	Suicidal			Helpless		
	(1)	(2)	(3)	(4)	(5)	(6)
$Damage_r$ × Post	0.0213**	0.0214**	0.0204**	0.0837***	0.0883***	0.0903***
	(0.0075)	(0.0076)	(0.0073)	(0.0203)	(0.0199)	(0.0187)
	[0.0048, 0.0378]	[0.0046, 0.0382]	[0.0044, 0.0365]	[0.0390, 0.1283]	[0.0446, 0.1320]	[0.0491, 0.1315]
Controls	No	No	Yes	No	No	Yes
Oblast FE	No	Yes	Yes	No	Yes	Yes
Survey FE	No	Yes	Yes	No	Yes	Yes
Mean of Dep. variable	0.0063	0.0063	0.0063	0.0991	0.0991	0.0991
Adj. R2	0.0095	0.0105	0.0118	0.0109	0.0238	0.0373
Observations	6148	6148	6148	6148	6148	6148

Notes: This table presents the results from the SUTVA problem test. We selected oblasts with war damage higher than $5 billion as those suffering from severe damage, including six oblasts: Donetska, Luhanska, Kharkivska, Kyivska, Zaporizka, and Chernihivska ($Damage_r$ = 1). Another six oblasts, which experienced the least damage (war damage between $0 and $0.2 billion), were grouped as the moderate war exposure group: Chernivtsi, Volynska, Ternopilska, Ivano-Frankivsk, Khmelnytsky, and Rivne ($Damage_r$ = 0). We reran the regression following model (1). Control variables include gender, age, age squared, education, nationality, and occupation. Variable definitions are provided in eTable 1. The abbreviation "FE" represents "Fixed Effects." Standard errors clustered at the oblast level are reported in parentheses, and 95% confidence intervals are shown in brackets. *, **, and *** indicate significance at the 10%, 5%, and 1% levels, respectively.

© 2025 An J et al. *JAMA Network Open*.

eTable 7. War and Civilians' Mental Health: Alternative Definition of War Intensity

Dep. variable	Suicidal			Helpless		
	(1)	(2)	(3)	(4)	(5)	(6)
$Damage_a$ × Post	0.0263***	0.0264***	0.0256***	0.0590*	0.0646*	0.0680**
	(0.0070)	(0.0073)	(0.0069)	(0.0297)	(0.0296)	(0.0280)
	[0.0097, 0.0429]	[0.0091, 0.0437]	[0.0093, 0.0418]	[-0.0114, 0.1293]	[-0.0053, 0.1346]	[0.0018, 0.1343]
Controls	No	No	Yes	No	No	Yes
Oblast FE	No	Yes	Yes	No	Yes	Yes
Survey FE	No	Yes	Yes	No	Yes	Yes
Mean of Dep. variable	0.0079	0.0079	0.0079	0.1072	0.1072	0.1072
Adj. R2	0.0061	0.0062	0.0075	0.0096	0.0300	0.0485
Observations	5458	5458	5458	5458	5458	5458

Notes: This table presents the results from the robustness tests using an alternative threshold definition of war intensity (from $5 billion to $10 billion) to determine whether an oblast suffers from severe or moderate war damage, following model (1). $Damage_a$ equals 1 for Donetska, Luhanska, Kharkivska, and Kyivska—the four regions that suffered at least $10 billion in losses—and equals 0 for Kirovohradska, Cherkaska, Poltavska, and Dnipropetrovska, which are the four oblasts that experienced moderate war damage but are adjacent to the severely damaged oblasts. Control variables include gender, age, age squared, education, nationality, and occupation. Variable definitions are provided in eTable 1. The abbreviation "FE" represents "Fixed Effects." Standard errors clustered at the oblast level are reported in parentheses, and 95% confidence intervals are shown in brackets. *, **, and *** indicate significance at the 10%, 5%, and 1% levels, respectively.

© 2025 An J et al. *JAMA Network Open*.

eTable 8. War and Civilians' Mental Health: Change Control Variables

**Panel A. Dep. Variable: Suicidal**						
	(1)	(2)	(3)	(4)	(5)	(6)
Damage × Post	0.0230***	0.0228***	0.0228***	0.0226***	0.0222***	0.0222***
	(0.0065)	(0.0065)	(0.0065)	(0.0064)	(0.0063)	(0.0063)
Female	-0.0002	0.0003	0.0003	0.0003	-0.0002	-0.0003
	(0.0013)	(0.0013)	(0.0013)	(0.0013)	(0.0013)	(0.0013)
Age		-0.0002	-0.0002	-0.0002		-0.0001
		(0.0004)	(0.0004)	(0.0004)		(0.0004)
Age squared		0.0000	0.0000	0.0000		-0.0000
		(0.0000)	(0.0000)	(0.0000)		(0.0000)
Primary education			0.0137	0.0169*	0.0156*	0.0181*
			(0.0084)	(0.0095)	(0.0087)	(0.0100)
Secondary education			0.0071***	0.0107	0.0127	0.0115
			(0.0012)	(0.0067)	(0.0079)	(0.0077)
Higher education			0.0067***	0.0104	0.0129	0.0120
			(0.0016)	(0.0070)	(0.0077)	(0.0073)
Ukrainian				-0.0068	-0.0050	-0.0061
				(0.0128)	(0.0138)	(0.0139)
Russian				-0.0123	-0.0105	-0.0117
				(0.0134)	(0.0144)	(0.0145)
Other nationality				-0.0082	-0.0065	-0.0077
				(0.0111)	(0.0120)	(0.0122)
Employed					-0.0079	-0.0082
					(0.0143)	(0.0143)
Self employed					-0.0040	-0.0036
					(0.0139)	(0.0140)
Other jobs					-0.0036	-0.0049
					(0.0143)	(0.0141)
Low finance						0.0049*
						(0.0026)
Mid finance						0.0026
						(0.0028)
Constant	0.0069***	0.0152	0.0080	0.0112	0.0048	0.0122
	(0.0008)	(0.0100)	(0.0104)	(0.0143)	(0.0071)	(0.0153)
Age FE	No	No	No	No	Yes	No
Oblast FE	Yes	Yes	Yes	Yes	Yes	Yes
Survey FE	Yes	Yes	Yes	Yes	Yes	Yes
Adj. R2	0.0041	0.0046	0.0045	0.0045	0.0029	0.0049
Observations	14140	14140	14140	14140	14138	14140

**Panel B. Dep. Variable: Helpless**						
	(1)	(2)	(3)	(4)	(5)	(6)
Damage × Post	0.0488**	0.0504**	0.0502**	0.0496**	0.0489**	0.0469**
	(0.0197)	(0.0198)	(0.0196)	(0.0194)	(0.0189)	(0.0180)
Female	0.0506***	0.0460***	0.0457***	0.0457***	0.0428***	0.0392***

© 2025 An J et al. *JAMA Network Open*.

	(0.0042)	(0.0041)	(0.0041)	(0.0041)	(0.0044)	(0.0040)
Age		0.0002	0.0006	0.0007		0.0011
		(0.0010)	(0.0009)	(0.0009)		(0.0009)
Age squared		0.0000	0.0000	0.0000		-0.0000
		(0.0000)	(0.0000)	(0.0000)		(0.0000)
Primary education			0.1264*	0.1495**	0.1342	0.1347*
			(0.0712)	(0.0677)	(0.0799)	(0.0784)
Secondary education			0.0264	0.0500	0.0464	0.0331
			(0.0276)	(0.0343)	(0.0419)	(0.0409)
Higher education			0.0268	0.0505	0.0478	0.0431
			(0.0262)	(0.0342)	(0.0417)	(0.0404)
Ukrainian				-0.0456	-0.0495	-0.0527
				(0.0414)	(0.0459)	(0.0455)
Russian				-0.0422	-0.0471	-0.0495
				(0.0455)	(0.0509)	(0.0494)
Other nationality				-0.0670	-0.0703	-0.0746
				(0.0409)	(0.0456)	(0.0450)
Employed					-0.0028	-0.0033
					(0.0517)	(0.0499)
Self employed					0.0051	0.0108
					(0.0523)	(0.0506)
Other jobs					0.0244	0.0115
					(0.0508)	(0.0492)
Low finance						0.0558***
						(0.0078)
Mid finance						0.0198***
						(0.0056)
Constant	0.0724***	0.0389*	0.0043	0.0263	0.0678**	0.0151
	(0.0026)	(0.0226)	(0.0321)	(0.0391)	(0.0255)	(0.0333)
Age FE	No	No	No	No	Yes	No
Oblast FE	Yes	Yes	Yes	Yes	Yes	Yes
Survey FE	Yes	Yes	Yes	Yes	Yes	Yes
Adj. R2	0.0346	0.0387	0.0394	0.0395	0.0410	0.0442
Observations	14140	14140	14140	14140	14138	14140

Notes: This table presents the robustness tests when changing control variables following model (1), including adding controls incrementally in a staggered approach from column (1) to column (4), absorbing age into the fixed effects in column (5), and further controlling for financial situations in column (6). Variable definitions are provided in eTable 1. The abbreviation "FE" represents "Fixed Effects." Standard errors clustered at the oblast level are reported in parentheses. *, **, and *** indicate significance at the 10%, 5%, and 1% levels, respectively.

© 2025 An J et al. *JAMA Network Open*.

eTable 9. Robustness Tests on Heterogeneous Impacts

Panel A. Dependent variable: Suicidal; *Post* × *Controls* included													
	Financial situation			Gender		Financial situation and gender						Education	
	(1)	(2)	(3)	(4)	(5)	(6)	(7)	(8)	(9)	(10)	(11)	(12)	(13)
	low	middle	high	female	male	low-female	low-male	middle-female	middle-male	high-female	high-male	low	high
Damage × Post	0.0176*	0.0073	0.0563***	0.0148***	0.0291**	0.0146*	0.0214	0.0083	0.0101	0.0401	0.0648**	0.0158	0.0312
	(0.0099)	(0.0071)	(0.0187)	(0.0052)	(0.0108)	(0.0083)	(0.0160)	(0.0113)	(0.0101)	(0.0335)	(0.0245)	(0.0115)	(0.011[illegible]
Controls	Yes	Yes	Yes	Yes	Yes	Yes	Yes	Yes	Yes	Yes	Yes	Yes	Yes
Oblast FE	Yes	Yes	Yes	Yes	Yes	Yes	Yes	Yes	Yes	Yes	Yes	Yes	Yes
Survey FE	Yes	Yes	Yes	Yes	Yes	Yes	Yes	Yes	Yes	Yes	Yes	Yes	Yes
Adj. R2	0.0136	0.0053	0.0121	0.0024	0.0128	0.0011	0.0402	0.0121	0.0027	0.0017	0.0242	0.0071	0.012[illegible]
Observations	6386	4516	2056	8174	5966	4183	2203	2429	2087	935	1121	8442	5620
Panel B. Dependent variable: Suicidal; *Damage* × *Controls* included													
	Financial situation			Gender		Financial situation and gender						Education	
	(1)	(2)	(3)	(4)	(5)	(6)	(7)	(8)	(9)	(10)	(11)	(12)	(13)
	low	middle	high	female	male	low-female	low-male	middle-female	middle-male	high-female	high-male	low	high
Damage × Post	0.0194*	0.0096	0.0555**	0.0161***	0.0303**	0.0140	0.0247	0.0110	0.0106	0.0369	0.0658**	0.0149	0.0329
	(0.0094)	(0.0069)	(0.0209)	(0.0050)	(0.0119)	(0.0083)	(0.0171)	(0.0115)	(0.0103)	(0.0298)	(0.0305)	(0.0108)	(0.012[illegible]
Controls	Yes	Yes	Yes	Yes	Yes	Yes	Yes	Yes	Yes	Yes	Yes	Yes	Yes
Oblast FE	Yes	Yes	Yes	Yes	Yes	Yes	Yes	Yes	Yes	Yes	Yes	Yes	Yes
Survey FE	Yes	Yes	Yes	Yes	Yes	Yes	Yes	Yes	Yes	Yes	Yes	Yes	Yes
Adj. R2	0.0050	0.0049	0.0072	0.0026	0.0083	0.0009	0.0224	0.0102	-0.0009	0.0002	0.0069	0.0046	0.009[illegible]
Observations	6386	4516	2056	8174	5966	4183	2203	2429	2087	935	1121	8442	5620
Panel C. Dependent variable: Helpless; *Post* × *Controls* included													
	Financial situation			Gender		Financial situation and gender						Education	
	(1)	(2)	(3)	(4)	(5)	(6)	(7)	(8)	(9)	(10)	(11)	(12)	(13)
	low	middle	high	female	male	low-female	low-male	middle-female	middle-male	high-female	high-male	low	high

© 2025 An J et al. *JAMA Network Open*.

Damage × Post	0.0398*	0.0007	0.1302**	0.0595***	0.0338	0.0570*	0.0124	-0.0025	0.0019	0.2796***	0.0421	0.0466**	0.049[illegible]
	(0.0207)	(0.0419)	(0.0533)	(0.0213)	(0.0423)	(0.0296)	(0.0464)	(0.0452)	(0.0620)	(0.0800)	(0.0538)	(0.0209)	(0.025[illegible]
Controls	Yes	Yes	Yes	Yes	Yes	Yes	Yes	Yes	Yes	Yes	Yes	Yes	Yes
Oblast FE	Yes	Yes	Yes	Yes	Yes	Yes	Yes	Yes	Yes	Yes	Yes	Yes	Yes
Survey FE	Yes	Yes	Yes	Yes	Yes	Yes	Yes	Yes	Yes	Yes	Yes	Yes	Yes
Adj. R2	0.0491	0.0453	0.0742	0.0463	0.0273	0.0508	0.0296	0.0468	0.0302	0.1027	0.0252	0.0438	0.051[illegible]
Observations	6386	4516	2056	8174	5966	4183	2203	2429	2087	935	1121	8442	562[illegible]
Panel D. Dependent variable: Helpless; *Damage* × *Controls* included													
	Financial situation			Gender		Financial situation and gender						Education	
	(1)	(2)	(3)	(4)	(5)	(6)	(7)	(8)	(9)	(10)	(11)	(12)	(13)
	low	middle	high	female	male	low-female	low-male	middle-female	middle-male	high-female	high-male	low	high
Damage × Post	0.0523**	0.0079	0.1151**	0.0657**	0.0303	0.0752**	0.0208	-0.0003	0.0066	0.2517***	0.0310	0.0451**	0.0558[illegible]
	(0.0216)	(0.0396)	(0.0466)	(0.0250)	(0.0409)	(0.0318)	(0.0465)	(0.0439)	(0.0597)	(0.0813)	(0.0523)	(0.0202)	(0.026[illegible]
Controls	Yes	Yes	Yes	Yes	Yes	Yes	Yes	Yes	Yes	Yes	Yes	Yes	Yes
Oblast FE	Yes	Yes	Yes	Yes	Yes	Yes	Yes	Yes	Yes	Yes	Yes	Yes	Yes
Survey FE	Yes	Yes	Yes	Yes	Yes	Yes	Yes	Yes	Yes	Yes	Yes	Yes	Yes
Adj. R2	0.0450	0.0377	0.0603	0.0398	0.0237	0.0452	0.0280	0.0423	0.0240	0.0943	0.0225	0.0421	0.042[illegible]
Observations	6386	4516	2056	8174	5966	4183	2203	2429	2087	935	1121	8442	562[illegible]

Notes: This table represents the robustness tests on heterogeneous effects of war damage on individuals' mental health following model (1), by financial situation, gender, financial situation × gender, and education. The dependent variable is *Suicidal* in Panel A and Panel B, and *Helpless* in Panel C and Panel D. Control variables include gender, age, age-squared, education, nationality and occupation. In Panel A and C, we further included the interaction terms between *Post* and control variables, and in Panel B and D, we further incorporated the interaction terms between *Severe* and control variables. Variable definitions are shown in eTable 1. Standard errors clustered at the oblast level are reported in parentheses. *, ** and *** indicate significance at the 10%, 5% and 1% levels, respectively.

© 2025 An J et al. *JAMA Network Open*.

eTable 10. Linking War to Mental Health Conditions

Panel A. War and Lost Job, Experience Bombing, Without a Livelihood, and Death of Loved

	(1)	(2)	(3)	(4)
Dependent variable	Lost job	Experience bombing	Without a livelihood	Death of loved
Damage × Post	0.097***	0.296***	0.040**	0.004
	(0.032)	(0.081)	(0.016)	(0.020)
	[0.032, 0.162]	[0.128, 0.463]	[0.007, 0.074]	[-0.037, 0.045]
Controls	Yes	Yes	Yes	Yes
Oblast FE	Yes	Yes	Yes	Yes
Survey FE	Yes	Yes	Yes	Yes
Mean of Dep. var	0.096	0.071	0.066	0.133
Adj. R2	0.059	0.310	0.026	0.029
Observations	14140	14140	14140	14140

© 2025 An J et al. *JAMA Network Open*.

eTable 11. Balanced Tests for Short-Term Analyses

Variables	Mean		Difference test of mean value	
	Before	Post	difference	p-value
Female	0.5408	0.5323	-0.0106	0.806
Age	44.7682	46.8954	2.1421	0.135
Secondary education	0.3691	0.3785	-0.0018	0.966
Higher education	0.6223	0.6215	0.0110	0.790
Ukrainian	0.9270	0.9262	0.0038	0.868
Russian	0.0258	0.0277	0.0007	0.959
Other nationality	0.0386	0.0369	-0.0046	0.786
Employed	0.5107	0.5138	0.0026	0.952
Self employed	0.1116	0.1169	0.0048	0.859
Other jobs	0.3691	0.3692	0.0019	0.964
Lost job	0.2060	0.2554	0.0473	0.185
Experience bombing	0.4549	0.5077	0.0566	0.157
Without a livelihood	0.0858	0.0954	0.0088	0.722
Death of loved	0.1888	0.2000	0.0152	0.655

Notes: This table shows the balance test results between respondents interviewed just before and after the Russian bombings, across a wide range of demographic characteristics and experiences, while controlling for settlement fixed effects.

© 2025 An J et al. *JAMA Network Open*.

eTable 12. War and Civilians' Mental Health in the Short Term

Dependent variable	Suicidal (mean: 0.0287)		Helpless (mean: 0.2204)		Lose faith in themselves (mean: 0.0645)	
	(1)	(2)	(3)	(4)	(5)	(6)
After	0.0272	0.0279	0.0552	0.0580	0.0459	0.0499
Robust SE	(0.0130)**	(0.0135)**	(0.0349)	(0.0347)*	(0.0202)**	(0.0204)**
	[0.0017, 0.0527]	[0.0013, 0.0545]	[-0.0134, 0.1237]	[-0.0102, 0.1261]	[0.0062, 0.0857]	[0.0098, 0.0900]
Settlement clustered SE	(0.0107)*	(0.0106)*	(0.0195)**	(0.0164)**	(0.0155)**	(0.0173)**
p-value (settlement bootstrapped clustered SE)	(0.074)*	(0.074)*	(0.000)***	(0.000)***	(0.000)***	(0.000)***
Individual characteristics	No	Yes	No	Yes	No	Yes
Settlement FE	Yes	Yes	Yes	Yes	Yes	Yes
Mean of Dep. var	0.0287	0.0287	0.2204	0.2204	0.0645	0.0645
Observations	558	558	558	558	558	558

Notes: This table presents the regression results for Suicidal, Helpless, and Lose faith in themselves regressed on After. Settlement fixed effects are included in all specifications, with individual characteristics controlled for in columns (2), (4), and (6). Individual characteristics include gender, age, age squared, education, nationality, and occupation. Robust standard errors, standard errors clustered at the settlement level, and p-values using settlement-bootstrapped clustered standard errors are reported in parentheses. Ninety-five percent confidence intervals for robust standard errors are shown in brackets. Variable definitions are provided in eTable 1. *, **, and *** indicate significance at the 10%, 5%, and 1% levels, respectively. The abbreviation "FE" represents "Fixed Effects," and "SE" represents "Standard Errors.".

© 2025 An J et al. *JAMA Network Open*.

Panel B. The impacts on *Suicidal*

Dependent variable	Suicidal					
	(1)	(2)	(3)	(4)	(5)	(6)
Damage × Post	0.022***	0.020***	0.019***	0.021***	0.022***	0.017**
	(0.006)	(0.006)	(0.006)	(0.007)	(0.006)	(0.006)
	[0.009, 0.036]	[0.007, 0.033]	[0.006, 0.032]	[0.008, 0.035]	[0.009, 0.036]	[0.004, 0.030]
Lost job		0.024***				0.019***
		(0.006)				(0.005)
		[0.012, 0.036]				[0.010, 0.029]
Experience bombing			0.012*			0.009
			(0.006)			(0.005)
			[-0.001, 0.025]			[-0.003, 0.020]
Without a livelihood				0.031***		0.026***
				(0.009)		(0.008)
				[0.012, 0.050]		[0.009, 0.042]
Death of loved					0.013***	0.012***
					(0.004)	(0.004)
					[0.004, 0.022]	[0.003, 0.020]
Controls	Yes	Yes	Yes	Yes	Yes	Yes
Oblast FE	Yes	Yes	Yes	Yes	Yes	Yes
Survey FE	Yes	Yes	Yes	Yes	Yes	Yes
Adj.R2	0.005	0.011	0.005	0.012	0.007	0.018
Observation	14140	14140	14140	14140	14140	14140

© 2025 An J et al. *JAMA Network Open*.

Panel C. The impacts on Helpless

Dependent variable	Helpless					
	(1)	(2)	(3)	(4)	(5)	(6)
Damage × Post	0.049**	0.041**	0.023	0.038**	0.049**	0.016
	(0.019)	(0.018)	(0.018)	(0.017)	(0.019)	(0.016)
	[0.009, 0.088]	[0.004, 0.078]	[-0.014, 0.060]	[0.004, 0.072]	[0.009, 0.088]	[-0.017, 0.049]
Lost job		0.078***				0.036***
		(0.010)				(0.013)
		[0.058, 0.099]				[0.010, 0.062]
Experience bombing			0.087***			0.065***
			(0.024)			(0.021)
			[0.037, 0.138]			[0.022, 0.108]
Without a livelihood				0.262***		0.249***
				(0.022)		(0.023)
				[0.216, 0.307]		[0.201, 0.297]
Death of loved					0.027***	0.019**
					(0.009)	(0.008)
					[0.009, 0.045]	[0.002, 0.037]
Controls	Yes	Yes	Yes	Yes	Yes	Yes
Oblast FE	Yes	Yes	Yes	Yes	Yes	Yes
Survey FE	Yes	Yes	Yes	Yes	Yes	Yes
Adj. R2	0.040	0.045	0.044	0.084	0.041	0.088
Observation	14140	14140	14140	14140	14140	14140

Notes: This table presents the DID results testing four mechanisms underlying the association between exposure to war and mental health: losing a job due to the war, experiencing bombings, living without a livelihood, and experiencing the death of loved ones. We controlled for individual characteristics, as well as oblast and survey fixed effects, following model (1). Individual controls include gender, age, age squared, education, nationality, and occupation. Variable definitions are provided in eTable 1. Standard errors clustered at the oblast level are reported in parentheses, and 95% confidence intervals are shown in brackets. *, **, and *** indicate significance at the 10%, 5%, and 1% levels, respectively.

© 2025 An J et al. *JAMA Network Open*.
