# Supplementary material for: Mental Health of Residents of Ukraine Exposed to the Russia-Ukraine Conflict
Source: JAMA Netw Open. 2025 Feb 13;8(2):e2459318. doi: 10.1001/jamanetworkopen.2024.59318 (PMC11826354; doi:10.1001/jamanetworkopen.2024.59318)
Supplement: Supplement 2. — Data Sharing Statement [file jamanetwopen-e2459318-s002.pdf]

Data Sharing Statement

An. Mental Health of Residents of Ukraine Exposed to the Russia-Ukraine Conflict. *JAMA Netw Open*. Published February 13, 2025. doi:10.1001/jamanetworkopen.2024.59318

Data
Data available: No
